# Effect of Long-Term Physical Exercise on Memory, Anxiety-Like Behavior, and Depression in Young and Aged Male Mice Subjected to Sleep Deprivation

**DOI:** 10.1055/s-0046-1829073

**Published:** 2026-10-19

**Authors:** Robson Salviano de Matos, Paulo Iury Gomes Nunes, Bruna Rafaele Diógenes da Silva, Daniel Vieira Pinto, Pedro Felipe Carvalhedo de Bruin, Reinaldo Barreto Oriá, Veralice Meireles Sales de Bruin

**Affiliations:** 1 Sleep and Biological Rhythms Laboratory, Federal University of Ceara (UFC), Fortaleza, Brazil; 2 Postgraduate Program in Medical Sciences, Federal University of Ceara (UFC), Fortaleza, Brazil; 3 Universidade Nilton Lins, Parque das Laranjeiras, Manaus, Brazil; 4 Laboratory of Wound Healing, Ontogeny, and Tissue Nutrition, Federal University of Ceara (UFC), Fortaleza, Brazil

**Keywords:** aerobic exercise, behavior, cognition, sleep disorder, sleep loss

## Abstract

This study evaluated whether chronic aerobic exercise could attenuate behavioral impairments associated with sleep deprivation (SD) in young and aged male mice. Young (3 months) and aged (18 months) Swiss mice were assigned to four groups: control, exercise (EXE), sleep deprivation (SD), and exercise followed by SD (EXE + SD). Animals performed treadmill training for 8 weeks and then underwent 72-hour SD. Behavioral tests assessed working memory (Y-maze), depression-like behavior (tail suspension), and anxiety-like behavior (elevated plus maze). The results suggest that age influences the effectiveness of exercise in mitigating sleep deprivation-induced behavioral deficits, opening avenues for future studies on the interaction between physical exercise, sleep, and aging.

## Introduction

It is well established that adequate sleep quality and duration are crucial for energy conservation, mood regulation, learning, memory consolidation, cognition, brain plasticity, and tissue repair. 1

In modern society, disturbances in sleep patterns, such as difficulty falling or staying asleep, increased daytime sleepiness, and reduced total sleep time, are increasingly recognized. Sleep deprivation (SD) is associated with physiological and pathological changes, causing both acute and chronic impairments in daily functioning and increasing health risks. 2

SD is defined as the reduction or partial suppression of sleep, and this condition disrupts cellular processes, negatively affecting behavior, neurobiology, and metabolism. 3

In response, strategies to counteract the harmful effects of SD have been increasingly investigated. The literature highlights that improving sleep hygiene and adopting healthier lifestyle habits may yield lasting benefits. 4 Regular physical exercise promotes cognitive and behavioral improvements; enhances brain health, mood, memory, sleep architecture; and contributes to overall quality of life. 5

Although studies confirm the beneficial effects of physical exercise on memory and on anxiety-like and depression-like behaviors in rodents, aging itself is associated with reduced neuroplasticity, impaired cognitive performance, and altered responses to stressors such as sleep loss. In parallel, sleep deprivation may differentially affect young and aged organisms, while the magnitude of exercise-induced protection may also vary according to age. 6 7 Therefore, evaluating young and aged mice under the same experimental conditions is important to determine whether the behavioral effects of chronic aerobic exercise in the context of sleep deprivation are age-dependent.

To the best of our knowledge, no study has comparatively investigated the effects of chronic aerobic exercise in rodent models of sleep deprivation across different age groups. Therefore, this study aimed to evaluate whether chronic physical exercise is associated with attenuation of SD-induced impairments in memory, anxiety-like behavior, and depression-like behavior in both young and aged mice.

## Materials and Methods

### Animals

This study was approved by the Animal Ethics Committee of the Federal University of Ceará (protocol number 8974250719). Eighty male Swiss mice were housed at 23  ±  2 °C under a 12-hour light/dark cycle with food and water available *ad libitum* . After weaning (21 days of age), animals were transferred to the laboratory and maintained under standard conditions until assignment to the young (3 months) or aged (18 months) cohorts. Each group was randomly assigned to one of four experimental conditions ( *n* = 10 per group; Fig. 1 ): Control, Exercise (EXE), Sleep Deprivation (SD), and Exercise–SD (EXE + SD).

**Fig. 1:**
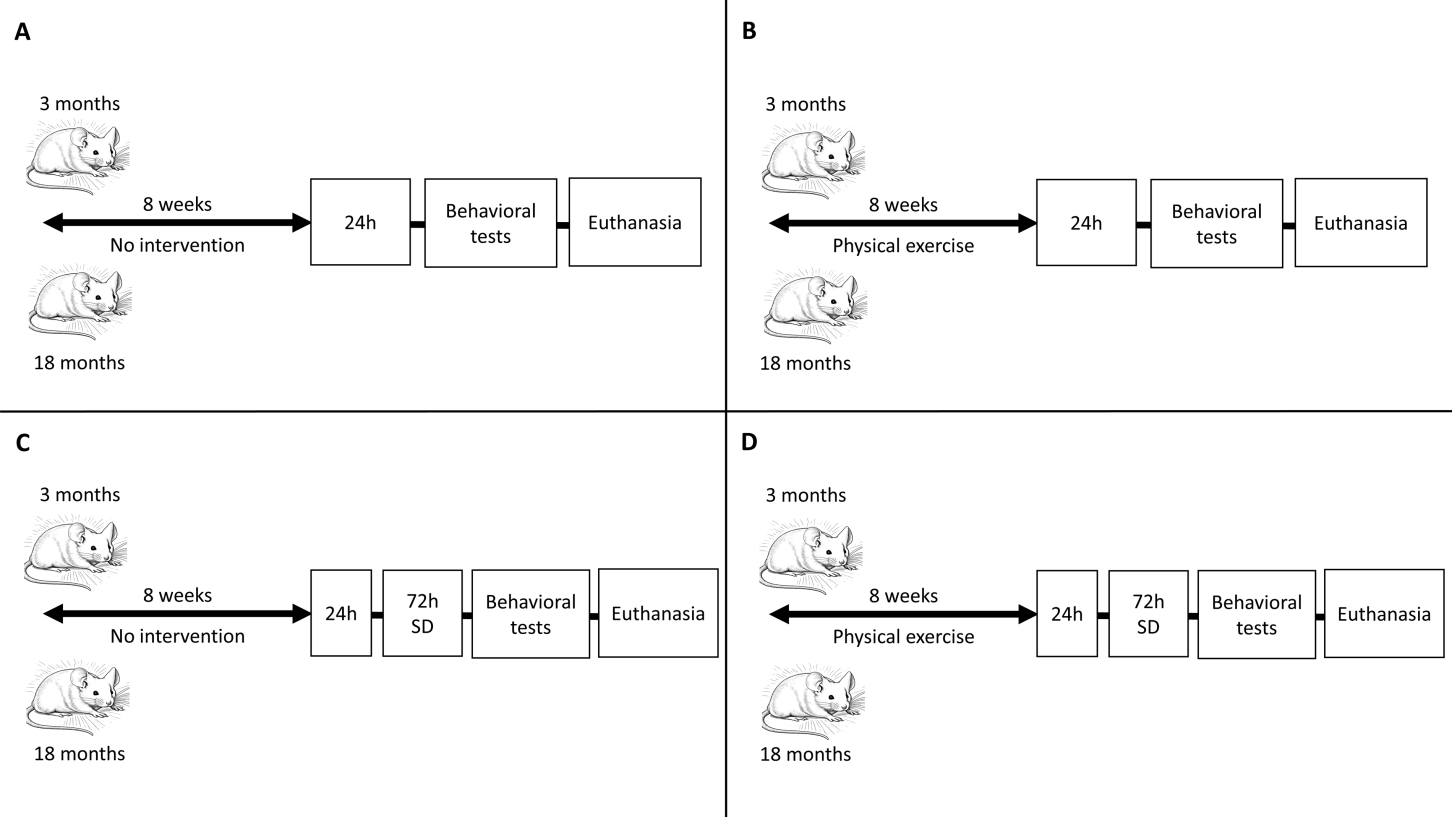
Study design for young and aged animals in the control condition (A), subjected to physical exercise (B), subjected to sleep deprivation only (C), and subjected to exercise prior to sleep deprivation (D), respectively. **Source:** Authors' own elaboration.

### Experimental Design

Sleep deprivation was induced over 72 hours using the modified multiple platform method, a protocol widely used to predominantly suppress paradoxical/REM sleep. The experiment was divided into two stages. In the first stage, young mice (3 months old; *n* = 40; 10 mice per cage) were used. In the second stage, aged mice (18 months old; *n* = 40; 10 mice per cage) were used. All animals were randomly distributed into four experimental groups ( *n* = 10 animals per group; *n* = 10 mice per cage): control, physical exercise (EXE; treadmill exercise for 8 weeks), sleep deprivation (SD; 72 hours of REM sleep deprivation [SD-REM]), and combination physical exercise + SD (EXE + SD; treadmill exercise for 8 weeks followed by 72 hours of SD-REM) ( Fig. 1 ). The behavioral tests were conducted after the completion of the protocols in each group, with a 5-minute interval between each test, in the following order: Y-maze, elevated plus maze, and tail suspension test. After the battery of behavioral tests, the animals were immediately euthanized using a guillotine.

### Exercise Protocol

Exercise sessions occurred from 11:00 to 13:00 on a motorized treadmill (Insight ^®^ ). After one week of adaptation (5 × 10 min, 3 m/min), animals performed a maximal aerobic capacity test. Training was moderate intensity (60% max speed), progressively increasing from 20 to 60 min/session over 8 weeks, with load adjustment after 4 weeks. 8 Electrical stimulation was not applied to the animals.

### Sleep Deprivation

SD was induced over 72 hours using the modified multiple platform method. 9 Groups of five mice were placed in water tanks with narrow platforms (3 cm diameter), spaced to prevent sustained sleep. Control and EXE groups were exposed to larger platforms (9 cm), allowing sleep.

### Behavioral Testing

Following the experimental protocols, animals underwent a sequence of behavioral tests to assess memory, depressive-like behavior, and anxiety-like behavior. Working memory was evaluated using the Y-maze, in which mice were placed in a maze with three identical arms. Spontaneous alternation behavior — defined as successive entries into different arms — was calculated as a percentage, along with the total number of entries, which reflects both cognitive performance and locomotor activity. 10 Depressive-like behavior was assessed through the tail suspension test, where mice were suspended by the tail for six minutes in an acoustically and visually isolated environment, and immobility time was recorded as an indicator of behavioral despair. 11 Anxiety-like behavior was evaluated using the elevated plus maze, consisting of two open and two closed arms elevated above the floor; mice were placed in the center, and both the number of entries and the time spent in open arms were measured over a five-minute period. Increased exploration of open arms indicates reduced anxiety, while preference for closed arms suggests heightened anxiety levels. 12 Because Y-maze performance may be influenced by emotional state, anxiety-like behavior was also assessed independently in the elevated plus maze ( Fig. 2 and Table 1 ).

### Statistical Analysis

Results are expressed as mean  ±  SEM. Data were tested for normality (Kolmogorov-Smirnov). Group comparisons were performed using one-way ANOVA or Kruskal-Wallis with Tukey's post hoc test, and unpaired t-test or Mann-Whitney test, as appropriate. Analyses were conducted using GraphPad Prism, version 5.03 (GraphPad Software, La Jolla, CA, USA); statistical significance was set at p < 0.05.

## Results

Baseline behavioral assessment (data not shown) revealed no statistically significant differences among groups in any of the investigated parameters.

**Table 1:** Behavioral data in young and aged mice

GROUPS	Y-Maze Test (Assessment of Working Memory)			
	Young Adult Animals		Aged Animals	
	Number of entries	% Spontaneous alternation	Number of entries	% Spontaneous alternation
Control	31.50 ± 1.94	64.13 ± 2.30	32.50 ± 0.62	59.89 ± 2.39
EXE	29.00 ± 1.53	64.04 ± 2.38	38.22 ± 1.33 ^d,g^	58.11 ± 2.53
SD	14.90 ± 1.79 ^a,b,d,e^	46.74 ± 6.24 ^a,b^	24.38 ± 1.38 ^a,b,d,e,g^	57.30 ± 3.85 ^g^
EXE + SD	24.57 ± 1.51 ^c,f^	68.10 ± 2.17 ^c,f^	26.50 ± 2.73 ^b,e^	60.86 ± 4.15
GROUPS	Tail Suspension Test (Assessment of Depression-Like Behavior)			
	Immobility Time after SD(s)			
	Young Adult Animals		Aged Animals	
Control	81.13 ± 6.69		69.43 ± 15.46	
EXE	59.43 ± 7.94		84.10 ± 11.63	
SD	127.10 ± 11.38 ^a,b,d,e^		138.2 ± 8.13 ^a,b,d,e^	
EXE + SD	68.63 ± 11.68 ^c,f^		72.29 ± 8.56 ^c,f^	
GROUPS	Elevated Plus Maze (Assessment of Anxiety-Like Behavior)			
	Young Adult Animals		Aged Animals	
	Number of entries	Time spent in open arms (sec.)	Number of entries	Time spent in open arms (sec.)
Control	6.92 ± 0.64	153.80 ± 10.06	9.50 ± 1.33	179.70 ± 5.99 ^g^
EXE	6.33 ± 0.55	161.10 ± 10.90	7.85 ± 0.82	172.10 ± 7.88
SD	4.00 ± 0.38 ^a,b,d,e^	87.00 ± 12.27 ^a,b,d,e^	8.62 ± 1.62 ^g^	111.90 ± 12.32 ^a,b,d,e^
EXE + SD	4.12 ± 0.64 ^a,d^	104.50 ± 19.81 ^b,e^	7.83 ± 1.64 ^g^	183.80 ± 23.67 ^c,f,g^

Numerical compilation of behavioral data. Descriptive data (mean ± SEM) of the evaluation of working memory by the Y-Maze test, depressive symptoms by the tail suspension test, and anxiety assessment by the elevated plus maze test in the Control, EXE, SD, and EXE+SD groups of young adult and aged mice. a *p* < 0.05 in relation to the Control group; b *p* < 0.05 in relation to the EXE group; and c *p* < 0.05 in relation to the SD group (ANOVA, Tukey post-test). d *p* < 0.05 in relation to the Control group; e *p* < 0.05 in relation to the EXE group; f *p* < 0.05 in relation to the SD group; and g *p* < 0.05 in relation to the corresponding group in young adult animals (unpaired t-test; Mann-Whitney test).

**Fig. 2:**
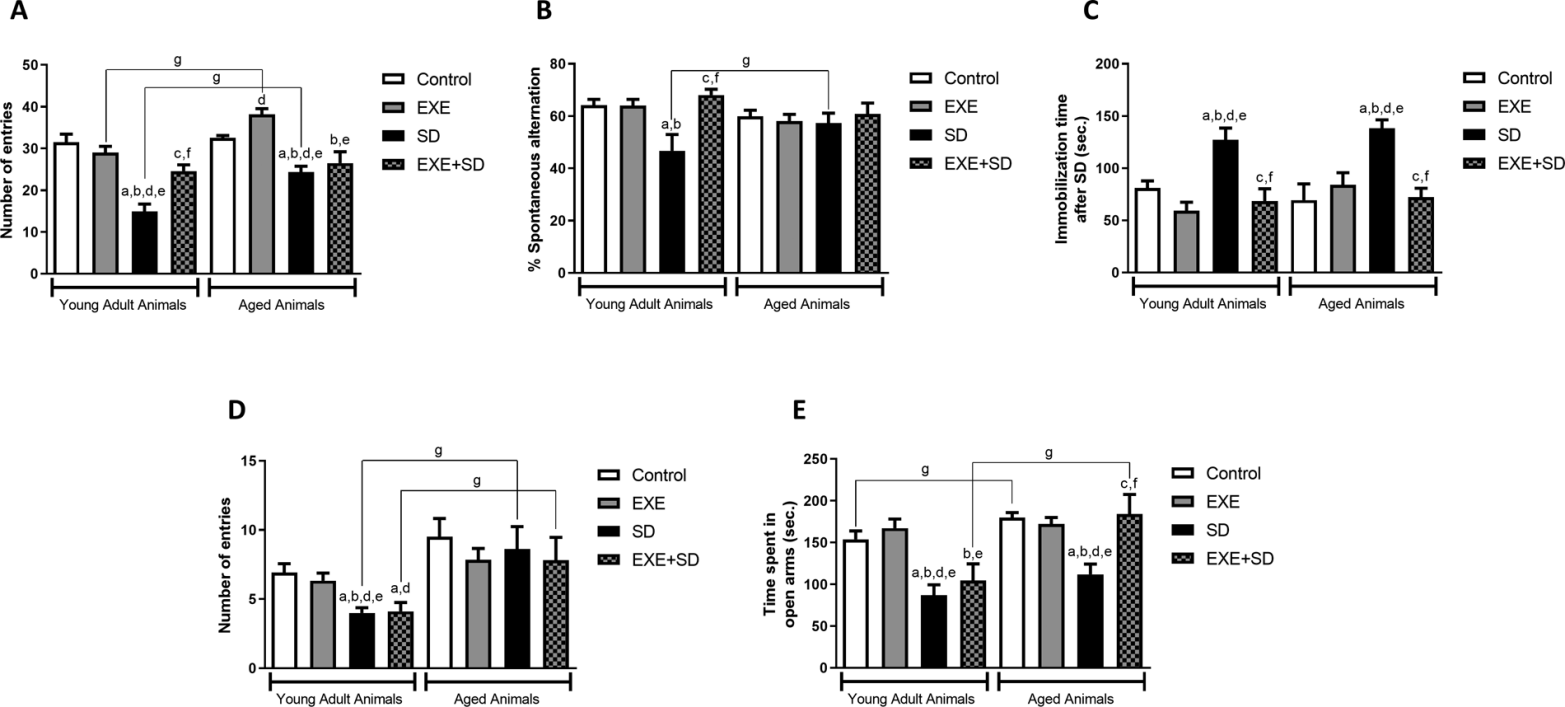
Graphs of behavioral evaluation of the Y-maze test ( **A** and **B** ), tail suspension test ( **C** ) and elevated plus maze test ( **D** and **E** ). Descriptive data (mean ± SEM) of the evaluation of working memory by the Y-Maze test ( **A** and **B** ), depressive symptoms by the tail suspension test ( **C** ), and anxiety assessment by the elevated plus maze test ( **D** and **E** ) in the Control, EXE, SD, and EXE+SD groups of young adult and aged mice. a *p* < 0.05 in relation to the Control group; b *p* < 0.05 in relation to the EXE group; and c *p* < 0.05 in relation to the SD group (ANOVA, Tukey post-test). d *p* < 0.05 in relation to the Control group; e *p* < 0.05 in relation to the EXE group; f *p* < 0.05 in relation to the SD group; and g *p* < 0.05 in relation to the corresponding group in young adult animals (unpaired t-test; Mann-Whitney test).

Working memory in young and aged animals was investigated using the Y-maze test. SD resulted in a significant reduction in the number of arm entries in the SD group compared with the Control and EXE groups in both young and aged animals. In young animals, SD was also associated with a reduction in the percentage of spontaneous alternations compared with the Control and EXE groups. In contrast, young animals previously exposed to exercise (EXE + SD) did not show the same reduction observed in the SD group, whereas this pattern was not observed in aged animals.

In the elevated plus maze, anxiety-like behavior was assessed by the number of entries into the open arms and by the time spent in the open arms. In young animals, SD reduced both the number of open-arm entries and the time spent in the open arms, indicating increased anxiety-like behavior. Prior exercise did not significantly attenuate these changes in the young EXE + SD group.

In aged animals, behavioral changes were observed only in the time spent in the open arms, with SD reducing this parameter, indicating increased anxiety-like behavior. Conversely, prior exercise was associated with preservation of time spent in the open arms in the EXE + SD group, which remained similar to the Control and EXE groups. In this test, aged animals showed higher open-arm entry counts and longer time spent in the open arms than the corresponding young groups.

The tail suspension test was employed as a measure of depression-like behavior, where both young and aged animals subjected to SD showed an increase in immobility time compared with the Control and EXE groups. Prior physical exercise before SD reduced immobility time in both age groups, and immobility time did not differ significantly between age groups.

## Discussion

To the best of our knowledge, this is the first study to examine the effects of chronic aerobic exercise on cognition and emotional-like behavioral outcomes in both young and aged sleep-deprived mice. In young animals, exercise attenuated memory impairment and depression-like behavior associated with SD. In aged mice, it attenuated anxiety-like and depression-like behavioral alterations, but did not improve memory performance.

The cognitive benefits of physical exercise are well documented and include increased cerebral blood flow, neurogenesis, and neurotrophic factor release, which support brain plasticity and preserve function with age. Conversely, sleep deprivation (SD) impairs processes such as spatial memory consolidation. 13

Consistent with prior findings, the present study shows that preemptive exercise attenuated SD-induced memory deficits in young mice. These effects are thought to result from modulation of oxidative stress and enhanced synaptic plasticity through regular physical activity. 14

In contrast, aged mice did not show the same memory-related response to exercise observed in young animals. This age-dependent difference may reflect neurostructural and functional changes associated with aging, including reduced neurogenesis, synaptic integrity, cerebrovascular adaptability, and network efficiency. Such alterations may disproportionately affect cognitive domains that depend on hippocampal integrity, while still allowing partial preservation of exercise responsiveness in affective-like behavioral measures. 15 Thus, the present findings suggest that the influence of exercise under sleep deprivation may be domain-specific in aged animals, with greater responsiveness in emotional-like than in memory-related outcomes.

Despite the lack of cognitive improvement, the absence of memory rescue does not invalidate the broader effects of exercise in aged animals. Rather, the present data indicate that age may shape which behavioral domains are more responsive to prior exercise under sleep deprivation. 16 From a practical perspective, these findings suggest that studies focusing exclusively on young animals may overestimate the generalizability of exercise-related cognitive protection, whereas inclusion of aged animals may reveal a more selective and translationally relevant response profile.

Regarding mood regulation, exercise effectively mitigated SD-induced depression-like behavior in both age groups. This aligns with evidence showing that physical activity enhances neurotransmitter release (e.g., serotonin and endorphins), promotes hippocampal neurogenesis, and modulates stress hormones such as cortisol. Additionally, neurotrophic signaling and reduced neuroinflammation likely contribute to the antidepressant effects of exercise. 17

Interestingly, SD altered anxiety-like behavior in both age groups, although the pattern and magnitude of the effect differed between young and aged animals, and exercise-related attenuation was more evident in aged mice. Young mice did not show significant changes, which is in line with previous studies. Conversely, other studies with young mice subjected to treadmill training and sleep deprivation reported reductions in anxiety-like behavior. 18 One possible explanation for the lack of change in anxiety-like parameters is the limitation of the model used in the present study. According to some authors, the increase in the number of entries and/or time spent in the open arms of adult animals may reflect hyperlocomotion or disinhibited behavior rather than a true anxiolytic-like effect. 19

In aged animals, the anxiolytic effects of exercise may be attributed to its regulatory influence on serotonin and GABA neurotransmission, both important for anxiety modulation. 20 These findings support the therapeutic potential of exercise in stress-related conditions associated with aging and sleep disruption.

One strength of the present study is the inclusion of two age groups, enabling age-dependent comparisons. The controlled conditions minimized mortality related to age, SD, and training. Limitations include the small set of behavioral tests, constrained by the need to minimize stress in SD animals, and the lack of a middle-aged group, which would enable a more comprehensive lifespan analysis. Additionally, longer-duration exercise in aged mice could yield more pronounced benefits. We also acknowledge that Y-maze performance is not exclusively determined by memory, since exploratory drive and emotional state may influence task behavior. For this reason, anxiety-like behavior was assessed separately in the elevated plus maze, allowing a more cautious interpretation of spontaneous alternation as a working memory-related outcome.

## Conclusion

The present results showed that SD impaired behavioral parameters and that chronic physical exercise attenuated working memory impairment and depression-like behavior in young adult mice, while reducing depression-like and anxiety-like alterations in aged mice. The present study reinforces the importance of chronic aerobic exercise as a low-cost, non-pharmacological intervention to attenuate working memory impairment and reduce depression-like and anxiety-like behavioral alterations. This is particularly relevant in challenging contexts such as SD, highlighting the complexity of the interactions among physical exercise, SD, and aging.
